# A Ground-Airborne Frequency-Domain Electromagnetic Rapid Imaging Method Based on Scalar Magnetic Field for Eliminating the Influence of Flight Attitude

**DOI:** 10.3390/s26165014

**Published:** 2026-08-07

**Authors:** Shuxu Liu, Zhongming Li, Yanfu Tang, Hongyu Li, Yongqiang Yang, Junlin Li

**Affiliations:** Changchun Institute of Optics, Fine Mechanics and Physics, Chinese Academy of Sciences, Changchun 130033, China; liushuxu@ciomp.ac.cn (S.L.); lizhongming@ciomp.ac.cn (Z.L.); tangyanfu@ciomp.ac.cn (Y.T.); lihongyu@ciomp.ac.cn (H.L.); yangyongqiang@ciomp.ac.cn (Y.Y.)

**Keywords:** underground structure detection, attitude noise, GAFEM, rapid imaging

## Abstract

**Highlights:**

**What are the main findings?**

- When the three-component magnetic field does not have a positive angle error, the proposed imaging method based on scalar magnetic fields eliminates, in principle, the influence of flight attitude on the ground-airborne frequency-domain electromagnetic (GAFEM) method.

**What are the implications of the main findings?**

- This work can achieve rapid and high-resolution imaging of underground anomalies in GAFEM detection where magnetic sensor attitude measurement capabilities are not available or the accuracy is poor, which is conducive to real-time field detection and the design of subsequent observation schemes.

**Abstract:**

The ground-airborne frequency-domain electromagnetic (GAFEM) method has the potential to detect underground anomalies at large depth ranges in areas with complex terrain. However, its specific configuration, which involves ground-based transmitting and airborne signal acquisition, inevitably introduces attitude noise into the measured magnetic field vector data. The presence of this attitude noise alters the characteristics of the measured data, consequently compromising the accuracy of imaging methods that rely on vector data. To eliminate the influence of attitude on GAFEM, this study proposes a rapid imaging technique for GAFEM based on the scalar magnetic field. This method utilizes the total magnetic field magnitude at each measurement point along the survey line as the input for imaging parameter calculation, thereby circumventing the effects of attitude noise and enabling high-resolution detection of underground anomalies. This study begins by analyzing the mechanism through which flight attitude affects GAFEM. Using a previously published GAFEM imaging method based on vector magnetic fields, it is demonstrated that flight attitude severely degrades the accuracy of such methods. Furthermore, a new imaging approach is proposed that employs the scalar magnetic field, which is immune to variations in flight attitude. A detailed description of the method’s principles, physical basis, and computational procedures is provided. The feasibility of the method is validated using a synthetic GAFEM model. The results indicate that the proposed method can achieve high-resolution detection of underground anomalies without being affected by attitude noise. Finally, the performance of the method is further tested using a simulation model constructed from actual geological data. The results confirm that the proposed method possesses the capability to identify underground anomalies in a complex model.

## 1. Introduction

The ground-airborne frequency-domain electromagnetic (GAFEM) method is a geophysical method capable of rapid underground structural investigation in complex terrain areas [1,2,3]. Within GAFEM research, rapid imaging methods, as a category of data interpretation methods, are valued for their fast imaging speed and low computational resource requirements, proving beneficial for field survey design and data quality assessment. The focus of GAFEM rapid imaging research has progressively shifted from the initial goal of simply detecting underground anomalies towards achieving high-resolution imaging. A typical trend is the development of various high-resolution imaging methods based on the classic apparent resistivity imaging (ARI) approach [4], such as the space magnetic gradient anomaly (SMGA) imaging method [5] and the tipper apparent resistivity-united-gradient imaging method [6].

Currently, high-resolution rapid imaging methods for GAFEM require the use of magnetic field components in one or multiple directions as fundamental imaging data. However, since the magnetic sensors in GAFEM are mounted on flight platforms, variations in flight attitude caused by wind direction during actual surveys introduce attitude noise into the measured magnetic field data. As the frequency of attitude noise coincides with that of the target signal, it is difficult to separate them completely through signal processing techniques [7,8], and the imaging results of high-resolution methods may exhibit significant deviations from the actual underground structure. Therefore, resolving the influence of attitude noise is of great importance for achieving high-resolution underground structure detection with GAFEM.

To reduce the influence of flight attitude on GAFEM, a rapid imaging method based on the scalar magnetic field is proposed in this study. This method enables the effective detection of underground anomaly location and boundaries by calculating the vector magnitude of the actual three-component magnetic field—specifically, the difference between the actual scalar magnetic field and the background scalar magnetic field. Scalar magnetic field data can be acquired through two approaches: 1. Using specialized magnetic sensors such as optically pumped magnetometers [9,10,11]. 2. Employing orthogonal tri-component sensors—including tri-component air-core coils and tri-component fluxgates—to simultaneously collect three-component magnetic field data [12,13], from which the total scalar field is derived by calculating the vector magnitude of the three components. Specialized scalar magnetic field sensors have not been widely applied in GAFEM, owing to limitations such as large physical dimensions and susceptibility to interference from the airborne platform. In contrast, the three-component sensor is commonly used as the magnetic field sensor in GAFEM; when the orthogonality among its three axes is sufficiently accurate, it is capable of realizing scalar magnetic field measurements that are independent of flight attitude. Therefore, when the three-component magnetic field sensor does not have a positive angle error, the proposed GAFEM rapid imaging method based on the scalar magnetic field theoretically eliminates the influence of flight attitude on GAFEM and achieves high-resolution imaging of underground electrical structures.

## 2. Analysis of the Influence of Flight Attitude on GAFEM

During the observational scheme design of GAFEM, a three-dimensional coordinate system is established based on the target magnetic field components, with the primary axis of the magnetic sensor aligned parallel to one axis of this coordinate system. However, during actual surveying, real-time variations in flight attitude caused by factors such as wind direction and flight platform performance lead to misalignment between the sensor’s primary axis and the reference coordinate axes [8]. Under such conditions, the magnetic field data acquired by the sensor represent the projection of the three-component magnetic field onto its primary axis. This recorded data incorporates both the target-direction magnetic field component and non-target-direction components. These non-target-direction components constitute what is referred to as attitude noise.

To analyze the influence of attitude noise on GAFEM, a parameter *δ* is first defined to quantify the deviation between the measured magnetic field data and the true value caused by flight attitude. The calculation method is shown in (1), where *B*_0_ represents the measured magnetic field when the sensor’s primary axis is aligned with the reference coordinate system (i.e., in the absence of attitude variation) and *B* denotes the measured magnetic field when the sensor’s primary axis is misaligned with the reference coordinate system due to attitude changes. Considering that the GAFEM system is currently only capable of measuring the magnitude of magnetic induction intensity with high precision, and in order to bring the present study closer to practical conditions, the magnetic field used in the analyses of this section and in the subsequent investigations is consistently the magnitude of magnetic induction intensity.

(1) $$\delta =\left|\frac{B-B_{0}}{B_{0}}\right|\times 100\%$$

**Figure 1:**
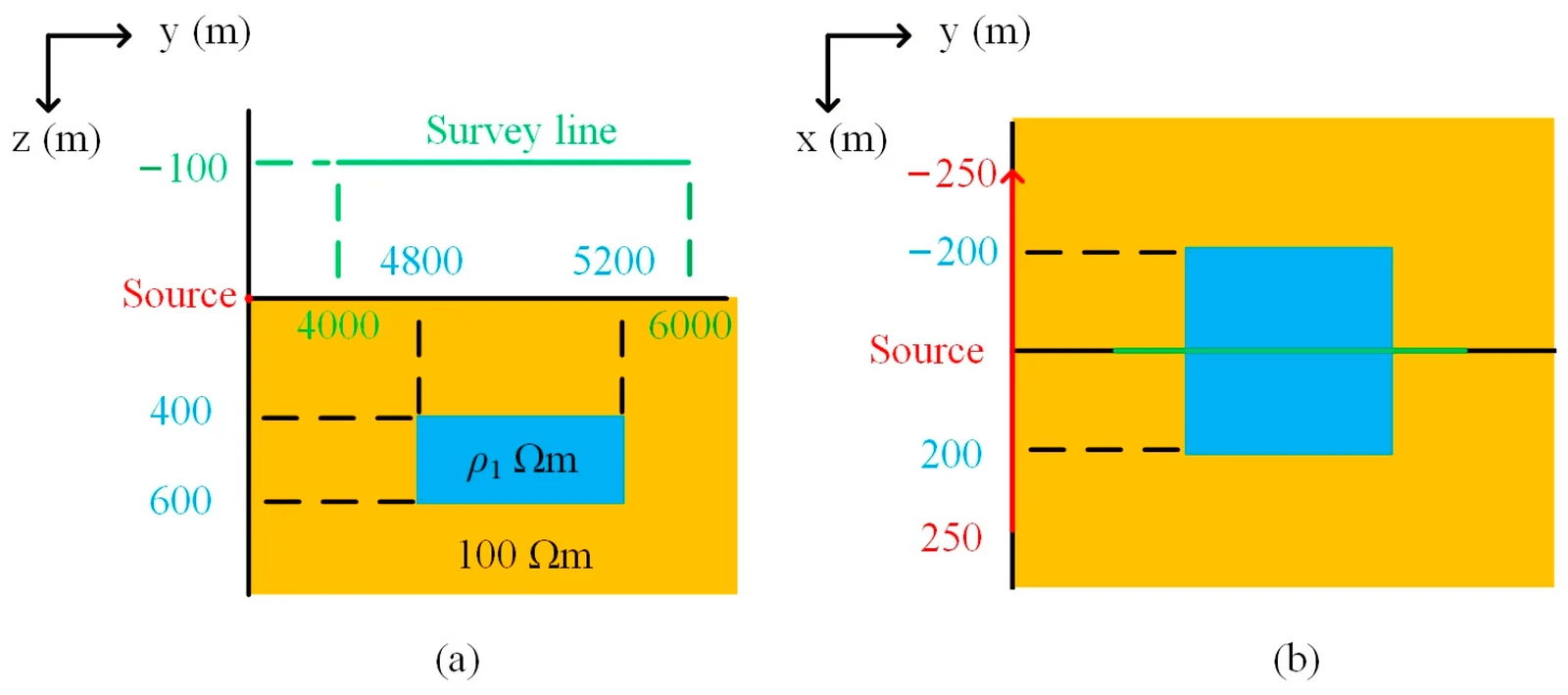
The GAFEM model: (**a**) yz profile; (**b**) xy profile.

Furthermore, a GAFEM model is established to analyze the influence of magnetic sensor attitude variations on measured magnetic field data, as shown in Figure 1. In the figure, the red line represents the transmitting source, with its center located at the coordinate origin. The transmitting current amplitude is 40 A. The yellow rectangle denotes the background earth while the blue rectangle indicates the anomalous body, with both positioned as shown in Figure 1. The resistivity of the anomaly is a low-resistivity anomaly (*ρ*_1_ = 10 Ωm). A survey line is defined along the straight path at *x* = 0 m, *y* = 4000 m to 6000 m, *z* = −100 m, represented by the green line in the figure. The parameters of each module of this model, such as the transmit-receive distance and the depth of the abnormal body, all meet the requirements of GAFEM for the remote area [5].

The electromagnetic fields for this model are computed using finite element software. The magnetic field along the survey line is extracted at 10 m intervals for different frequencies as the measured magnetic field without attitude variation, *B*_0_. The attitude angles of GAFEM are essentially the angles between the detection coordinate system *O*-*xyz* and the geographic coordinate system *O*-*x*_0_*y*_0_*z*_0_. Depending on the coordinate axis about which the rotation is performed, the attitude angles are defined in the following three ways:

(1) Yaw angle *ψ*: The angle between the projection of the x-axis onto the *x*_0_*Oy*_0_ plane and the *x*_0_-axis, positive when yawing to the right. The yaw angle is rotated about the *z*_0_-axis.

(2) Pitch angle *θ*: The angle between the projection of the x-axis onto the *x*_0_*Oz*_0_ plane and the *x*_0_-axis, positive when pitched upward. The pitch angle is rotated about the y_0_-axis.

(3) Roll angle *γ*: The angle between the projection of the y-axis onto the *y*_0_*Oz*_0_ plane and the *y*_0_-axis, positive when rolling to the right as viewed along the heading direction. The roll angle is rotated about the *x*_0_-axis.

In an ideal situation, *ψ* = *θ* = *γ* = 0°. The measured three-component magnetic field of GAFEM is *B*_0_ = (*B_x_*_0_, *B_y_*_0_, *B_z_*_0_). After the change in attitude angles, the magnetic field becomes *B* = (*B_x_*, *B_y_*, *B_z_*). The two satisfy the relationship as shown in (2).

(2) $$\left[\begin{array}{c} B_{x}\\ B_{y}\\ B_{z} \end{array}\right]=R\left[\begin{array}{c} B_{x_{0}}\\ B_{y_{0}}\\ B_{z_{0}} \end{array}\right]=R_{R}R_{P}R_{Y}\left[\begin{array}{c} B_{x_{0}}\\ B_{y_{0}}\\ B_{z_{0}} \end{array}\right]$$

*R* is the full rotation matrix and *R_R_*, *R_P_* and *R_Y_* are the rotation matrices of roll, pitch and yaw angles, respectively.

(3) $$R_{R}=\left[\begin{array}{ccc} 1 & 0 & 0\\ 0 & \cos\gamma & \sin\gamma \\ 0 & -\sin\gamma & \cos\gamma \end{array}\right]$$

(4) $$R_{P}=\left[\begin{array}{ccc} \cos\theta & 0 & -\sin\theta \\ 0 & 1 & 0\\ \sin\theta & 0 & \cos\theta \end{array}\right]$$

(5) $$R_{Y}=\left[\begin{array}{ccc} \cos\psi & \sin\psi & 0\\ -\sin\psi & \cos\psi & 0\\ 0 & 0 & 1 \end{array}\right]$$

According to the matrix multiplication algorithm, the full rotation matrix *R* can be obtained:

(6) $$R=\left[\begin{array}{ccc} \cos\theta \cos\psi & \cos\theta \sin\psi & -\sin\theta \\ \sin\gamma \sin\theta \cos\psi -\cos\gamma \sin\psi & \sin\gamma \sin\theta \sin\psi +\cos\gamma \cos\psi & \sin\gamma \cos\theta \\ \cos\gamma \sin\theta \cos\psi +\sin\gamma \sin\psi & \cos\gamma \sin\theta \sin\psi -\sin\gamma \cos\psi & \cos\gamma \cos\theta \end{array}\right]$$

**Figure 2:**
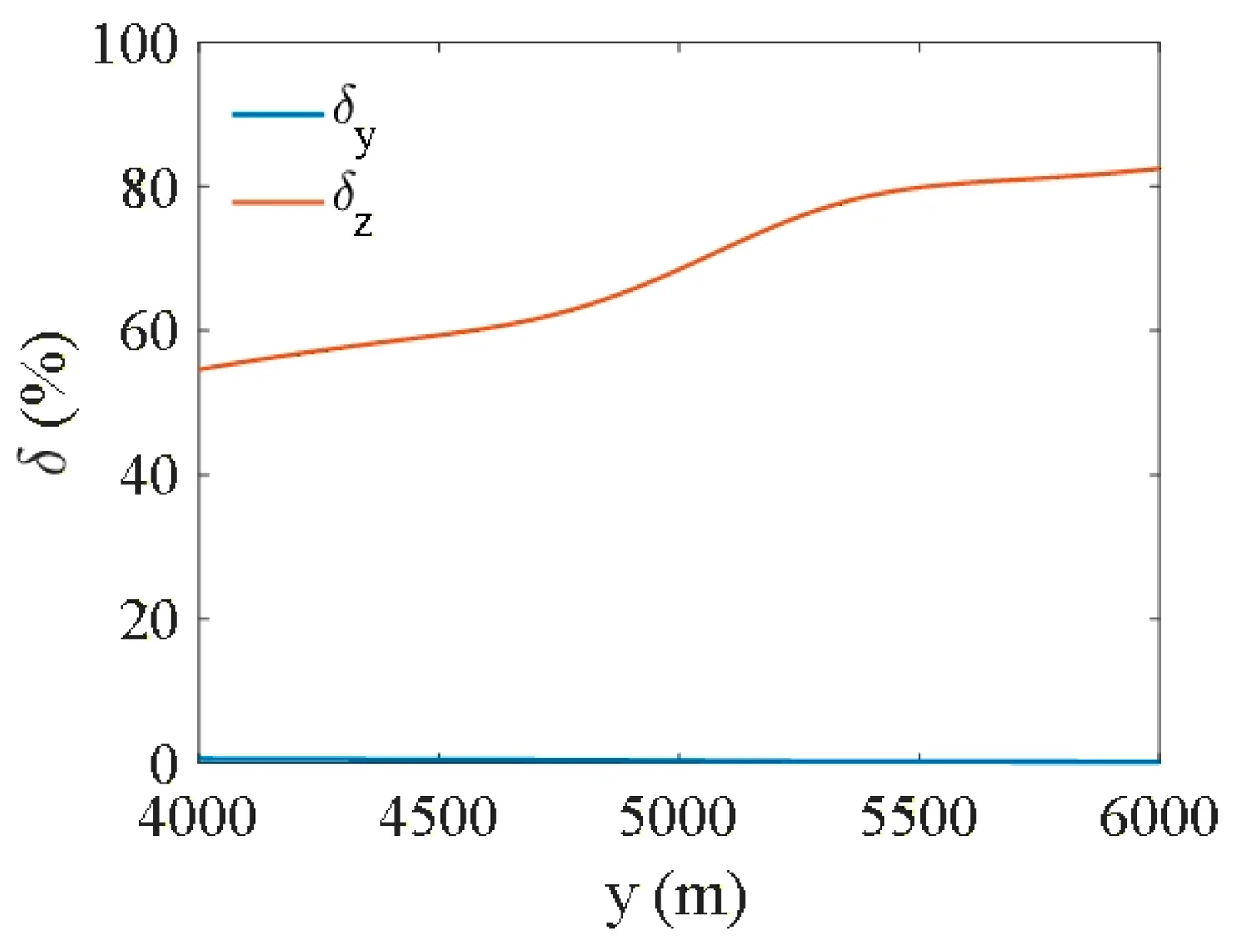
The deviation between measured magnetic field data and true values along the survey line caused by flight attitude.

Using (2) and (6), the measured magnetic field under attitude variation *B* can be obtained [8]. The roll angle *γ*, pitch angle *θ*, and yaw angle *ψ* are all set to 5°. Using (1), *δ* along the survey line is calculated, with the resulting curves plotted in Figure 2. The analysis utilizes magnetic field data at a frequency of 128 Hz. Due to the configuration of the transmitting source in the model shown in Figure 1, the *x*-component along this survey line becomes negligible. Therefore, the *y*-component and *z*-component of the magnetic field are selected as the subjects of investigation in Figure 2.

As observed from the Figure, when the magnetic sensor’s attitude changes, a significant deviation is observed between the measured magnetic field *B_z_*_0_ and its true value, whereas the deviation of *B_y_*_0_ remains negligible. The deviation *δ_z_* increases with the transmitting-receiving distance, reaching a maximum of 82.5%. This phenomenon is attributed to the fact that in GAFEM, the lateral component of the measured magnetic field is 1–2 orders of magnitude greater than the vertical component. Moreover, with increasing transmitting-receiving distance, the attenuation rate of the lateral component is significantly slower than that of the vertical component. When flight attitude variations occur, the lateral component measurement incorporates a coupled contribution from the vertical component, and vice versa. Due to the substantially larger magnitude of the lateral component, its measurement is minimally affected by the vertical component, and this influence further diminishes with increasing transmitting-receiving distance. In contrast, the vertical component measurement is severely affected by the coupled lateral component, and this interference intensifies with growing transmitting-receiving distance. These results clearly demonstrate that the vertical component of the measured magnetic field is more vulnerable to flight attitude variations compared to the lateral component. When the vertical component measurements contaminated with attitude noise are utilized as raw data for imaging, the resulting images suffer from severe distortion and may completely lose their capability to identify underground anomalies.

**Figure 3:**
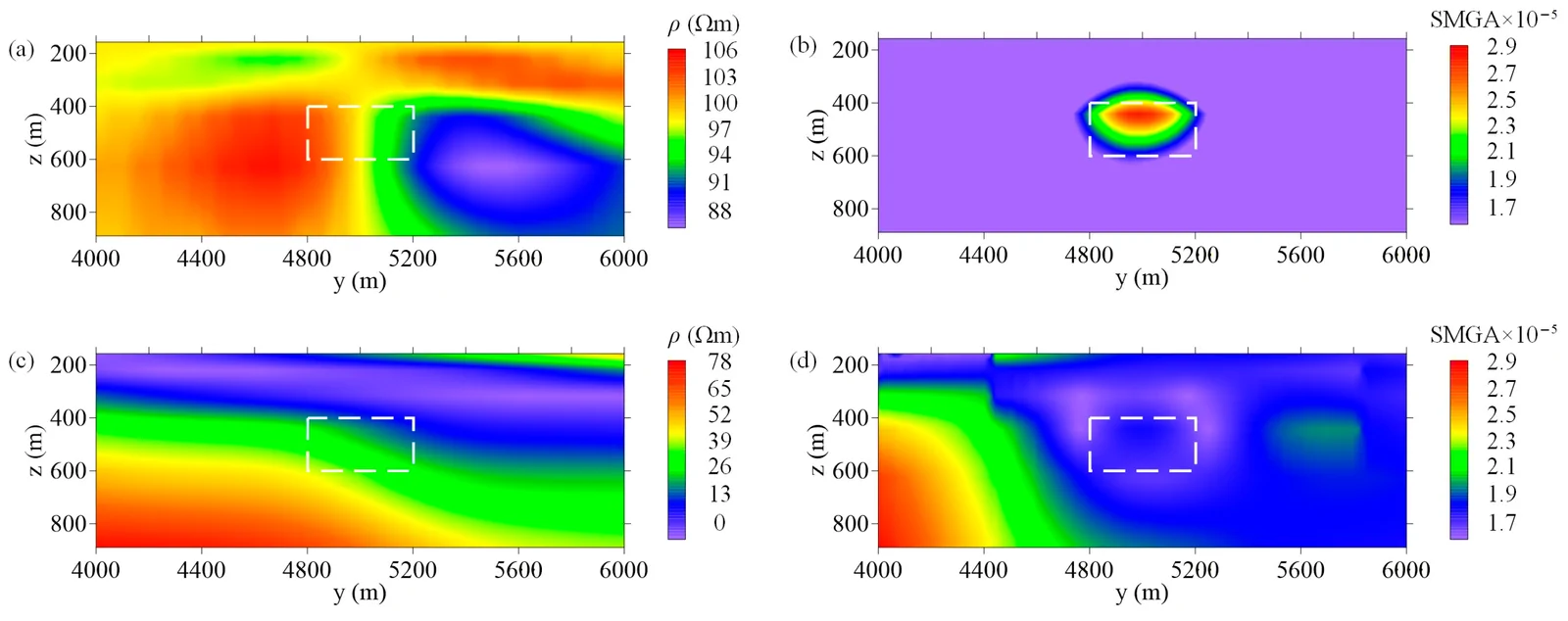
Imaging results of the model in Figure 1 using ARI and SMGA methods under different conditions. Without attitude noise: (**a**) imaging result of ARI; (**b**) imaging result of SMGA. With attitude noise: (**c**) imaging result of ARI; (**d**) imaging result of SMGA.

To further illustrate the influence of flight attitude on GAFEM, imaging of the model in Figure 1 is performed using both ideal magnetic field data (directly obtained from finite element software) and attitude-noise-contaminated magnetic field data (calculated from the ideal data using (2) and (6)), with the results presented in Figure 3. The dashed lines in Figure 3 indicate the actual positions and boundaries of the underground anomalies. The magnetic field data used in the imaging process employ logarithmically spaced frequencies commonly utilized in practical surveys, with a range of 32–1024 Hz. The logarithmic frequency interval is adopted owing to the common use of a 2^n^ pseudo-random waveform as the transmitted signal in field surveys, for which the dominant frequency intervals are logarithmically spaced. Furthermore, for frequency-domain electromagnetic detection, based on the calculation method of the frequency-domain skin depth, it can be deduced that, for the model illustrated in Figure 1, if the target detection depth is 200–800 m, the lower limit of the transmission frequency must be no greater than 40 Hz, and the upper limit no less than 625 Hz. Therefore, in conjunction with the commonly used frequencies of the 2^n^ pseudo-random waveform, the transmitting frequency range is set to 32–1024 Hz.

As observed in Figure 3a,b, both ARI and SMGA can effectively identify underground anomalies when using ideal data. Specifically, ARI locates underground anomalies through the transition zone between low and high apparent resistivity in the imaging results. In Figure 3a, the central position of the transition zone aligns with the center of the anomaly. Furthermore, based on ARI’s characteristic capability for identifying anomaly conductivity types, the transition from high resistivity at the source-proximal end to low resistivity at the source-distal end in Figure 3a indicates a low-resistivity anomaly, which is consistent with the configuration of the model in Figure 1. SMGA identifies underground anomalies by detecting the positional characteristics of anomalous regions in the imaging results. In Figure 3b, an anomalous region is observed whose location and boundaries correspond to those of the anomaly in Figure 1. Additionally, according to SMGA’s characteristic for identifying anomaly conductivity types, when the anomalous region exhibits positive values, it signifies a low-resistivity anomaly, which also conforms to the model configuration in Figure 1.

As observed in Figure 3c,d, when using attitude-noise-contaminated data, significant alterations occur in the imaging results of both methods, rendering them essentially incapable of identifying underground anomalies according to their respective methodological definitions. In the SMGA imaging results, the anomalous region is concentrated in the deep section near the transmitter, which does not correspond to the anomaly position in Figure 1. Figure 3d demonstrates that attitude noise severely interferes with SMGA, fundamentally impairing its ability to effectively identify underground anomalies.

## 3. Imaging Method Based on Scalar Magnetic Anomaly Gradient

Section 2 demonstrates that variations in the flight attitude of the magnetic sensor can seriously affect the accuracy of magnetic field vector-based imaging methods in GAFEM. Current attitude measurement systems and attitude correction algorithms are insufficient to fully meet the requirements of GAFEM in complex surveying environments. Therefore, to enhance the practical applicability of GAFEM in field detections, it is necessary to investigate methodological approaches to reduce or eliminate the influence of attitude noise from a theoretical perspective.

To mitigate or eliminate the influence of attitude noise on GAFEM from a methodological perspective, the most effective approach is to employ data that is minimally affected or entirely unaffected by flight attitude as the basic data for imaging. Among the currently measurable data in GAFEM, the scalar magnetic field *B_S_* at airborne survey points satisfies this requirement. Calculation of the vector magnitude from three-component magnetic field data acquired with tri-component sensors (e.g., tri-component fluxgate sensors) is used to obtain *B_S_*, with the computational method shown in (7) [12].

(7) $$B_{S}\left(i,f_{k}\right)=\sqrt{B_{x}^{2}\left(i,f_{k}\right)+B_{y}^{2}\left(i,f_{k}\right)+B_{z}^{2}\left(i,f_{k}\right)}$$

In (4), *B_x_*(*i*, *f_k_*), *B_y_*(*i, f_k_*), and *B_z_*(*i, f_k_*) represent the *x*, *y*, and *z* components of the magnetic field, respectively, at each survey point *i* (*i* = 1, 2…*p*, *p* is the total number of measurement points on a measurement line) along the survey line with frequency *f_k_*. (*k* = 1, 2…*q*, *q* is the total number of transmitting frequencies) During measurement using this method, attitude variations cause simultaneous changes in the acquired three-component magnetic field data. However, the total magnetic induction intensity at the sensor location remains unaffected by attitude changes. Consequently, the value of *B_S_* calculated using (7) is also immune to the effects of attitude variations.

Based on the research foundation of SMGA, it is established that the difference between the actually measured magnetic field and the background magnetic field without underground anomalies can effectively identify underground anomalies. To enable the scalar magnetic field with effective underground anomaly detection capability, an imaging apparent parameter based on the scalar magnetic field is defined, termed the scalar magnetic anomaly (SRMA), with its calculation method shown in (8).

(8) $$SRMA\left(i,f_{k}\right)=\frac{B_{S}\left(i,f_{k}\right)-B_{S-BG}\left(i,f_{k}\right)}{B_{S-BG}\left(i,f_{k}\right)}$$

In the equation, *B_S_*_-*BG*_ represents the background magnetic field. In practical surveys, *B_S_*_-*BG*_ cannot be directly measured. According to research by Liu et al., the background resistivity of the survey area, when fitted using *B_S_*_-*BG*_, achieves high accuracy [14]. In such cases, an equivalent survey model without underground anomalies can be constructed based on the known transmitting source, survey area information, and the fitted background resistivity. The *B_S_*_-*BG*_ field is then obtained through 3D forward modeling. Therefore, SRMA is a dimensionless parameter that merely represents the degree of change in the measured scalar magnetic field relative to the background scalar magnetic field. Furthermore, based on SMGA, gradient fields provide higher resolution in detecting underground anomalies than absolute fields. Following this principle, the scalar magnetic anomaly gradient (SRMAG) is defined based on SRMA, as shown in (9).

(9) $$SRMAG(i,f_{k})=\left\{\begin{array}{ll} \frac{SRMA(i,f_{2})-SRMA(i,f_{1})}{\log_{10}f_{2}-\log_{10}f_{1}} & \left(k=1\right)\\ \frac{SRMA(i,f_{k+1})-SRMA(i,f_{k-1})}{2(\log_{10}f_{k+1}-\log_{10}f_{k-1})} & \left(1<k<q\right)\\ \frac{SRMA(i,f_{q})-SRMA(i,f_{q-1})}{\log_{10}f_{q}-\log_{10}f_{q-1}} & \left(k=q\right) \end{array}\right\}$$

In (6), SRMAG is determined by calculating the ratio of the difference in logarithmic frequency values (i.e., log_10_*f_k_*) at different frequencies at the same measurement point to the difference in SRMA. The unit of SRMAG is /log_10_(Hz), which represents the degree of change in SRMA caused by variations in frequency. SRMAG is a parameter related to both survey point position and frequency. During imaging, SRMAG needs to be projected to target underground positions based on survey point locations and frequencies. Equation (10) provides the calculation method for projecting the apparent parameter to underground positions during imaging.

(10) $$\begin{array}{l} x=X(i)\\ y=Y(i)\\ z=\sqrt{\rho_{0}/(\pi f_{k}\mu )} \end{array}$$

In the equation, the horizontal coordinates (*x*,*y*) are determined by the horizontal position of the survey point (*X*(*i*), *Y*(*i*)). The depth *z* is calculated using the frequency-domain skin depth. In (10), *ρ*_0_ represents the background resistivity and *μ* denotes the background permeability. For pure electrical model studies, *μ* is substituted with the vacuum permeability. Through (10), SRMAG can be correlated with the imaged underground positions.

**Figure 4:**
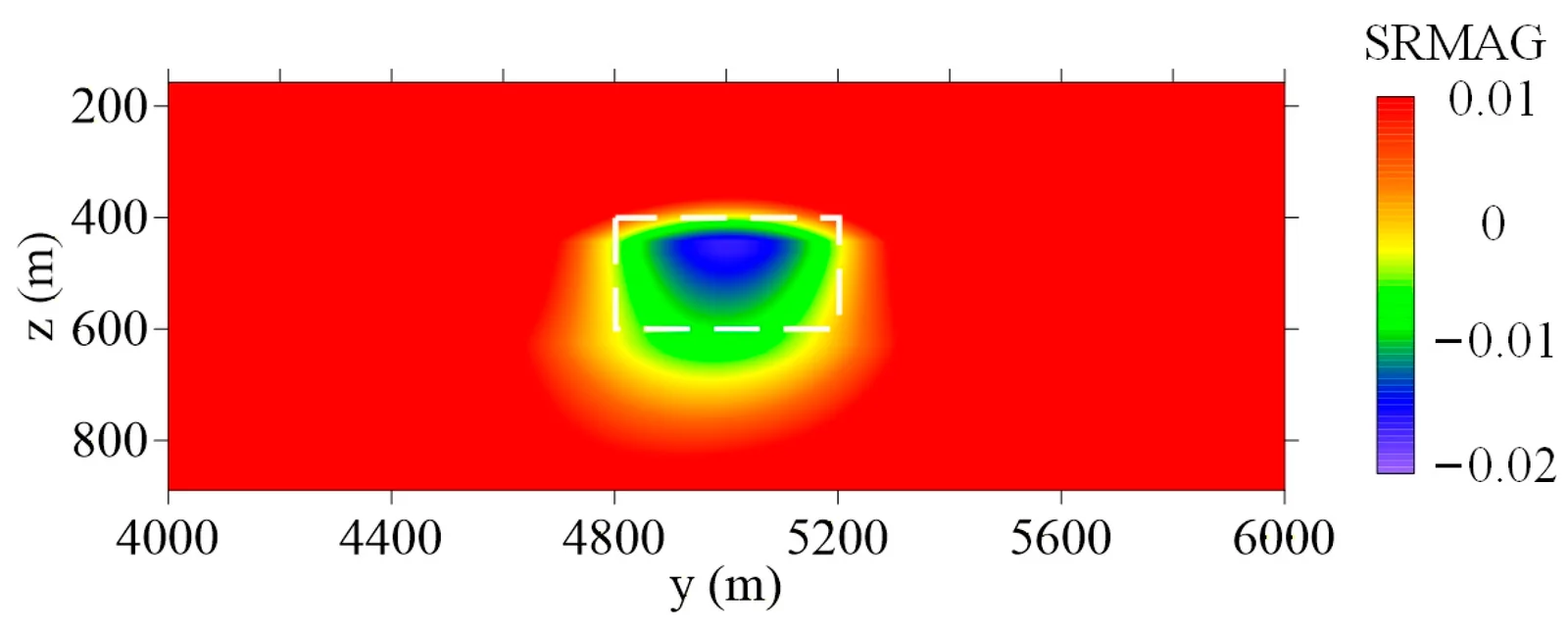
Imaging results of the model in Figure 1 using SRMAG.

To evaluate the underground detection capability of SRMAG, imaging of the model in Figure 1 is conducted again using this method to verify its effectiveness, with the imaging results presented in Figure 4. Among them, when obtaining the three-component magnetic field data, it is assumed that there is no error in the orthogonality.

Figure 4 reveals anomalous zones similar to those identified through SMGA imaging. The positions and boundaries of these anomalous zones demonstrate substantial consistency with the anomaly configuration in the model shown in Figure 1. Based on the imaging results presented in Figure 4, SRMAG is demonstrated to achieve high-resolution detection of both the positions and boundaries of underground anomalies.

To evaluate the influence of flight attitude on the scalar magnetic field-based SRMAG, the ideal data used in Figure 4 are further processed by incorporating attitude noise through (2), simulating tri-axial attitude angles with random variations between 1° and 5° along the entire survey line. The imaging results obtained under these conditions remain entirely consistent with those presented in Figure 4. This confirms that SRMAG remains unaffected by flight attitude variations.

**Figure 5:**
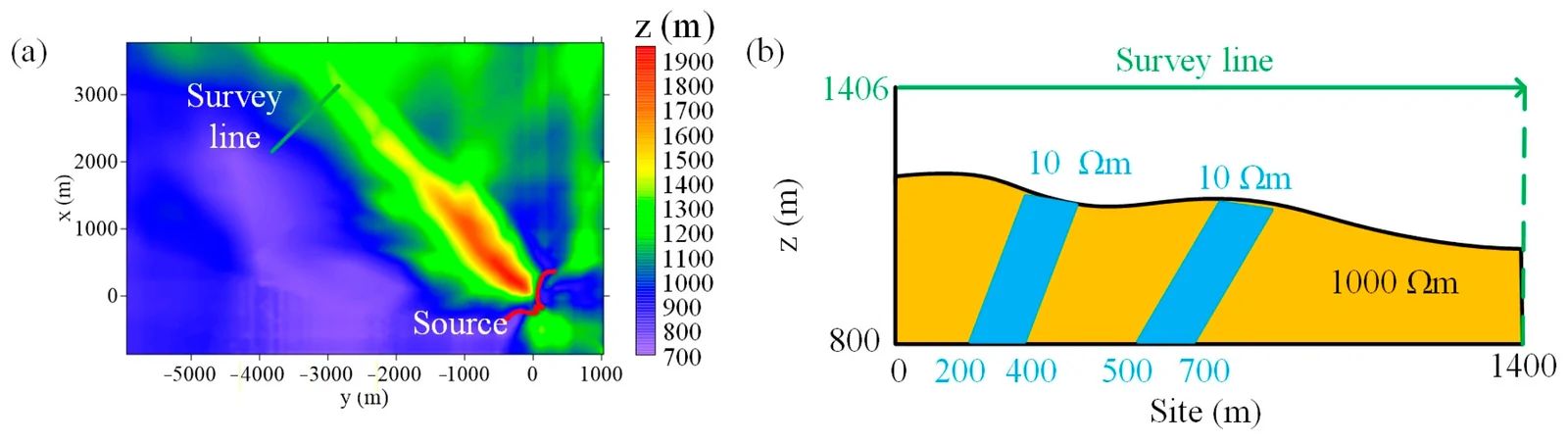
GAFEM simulation model. (**a**) Terrain distribution of the survey model. (**b**) Vertical profile where the survey line is located.

To further validate the performance of SRMAG, an equivalent model is established based on topographic data from a specific location in China, with designed transmitting sources and survey lines as shown in Figure 5.

**Figure 6:**
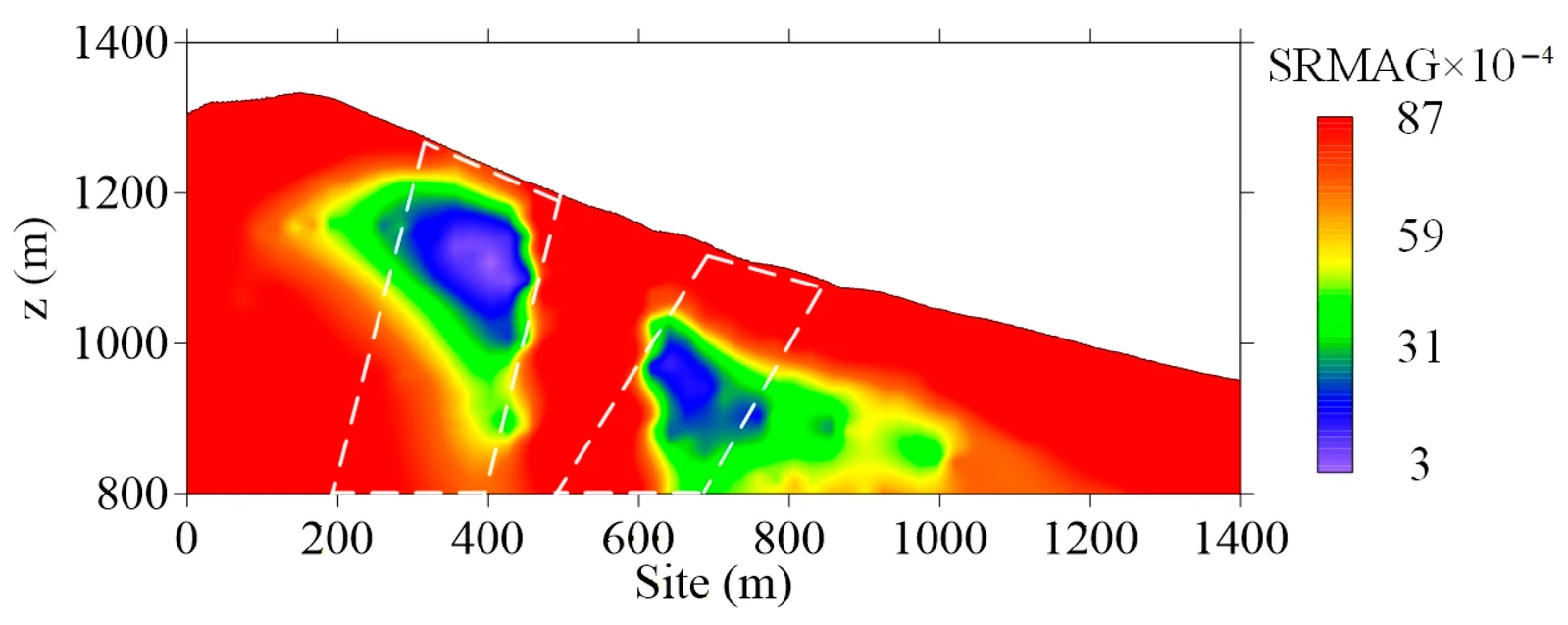
Imaging results of the model in Figure 5 using SRMAG.

Figure 5a displays the elevation distribution of the entire survey model, including the transmitting source, where the red line represents the transmitting source. The transmitting frequencies employ logarithmically spaced frequencies ranging from 32 Hz to 8192 Hz. Figure 5b shows the vertical profile along the survey line, with the horizontal coordinate indicating the distance of each survey point relative to the starting point of the line. The yellow area represents the background earth, while the blue areas indicate underground anomalies. The earth resistivity is set to 1000 Ωm, with both anomalous bodies configured as low-resistivity anomalies at 10 Ωm. Using the green line in Figure 5 as the survey line, the three-component magnetic field at each survey point along the line is computed at 10 m intervals, and attitude noise is incorporated using (2) to simulate GAFEM acquisition data. During the attitude noise incorporation, tri-axial attitude angles vary randomly between 1° and 5° along the entire survey line, and it is believed that there is no error in the orthogonality of the three-component magnetic field directions. The model in Figure 5 is imaged using SRMAG, with the imaging results presented in Figure 6.

Figure 6 reveals anomalous zones similar to those observed in Figure 4. The positions of these anomalous zones correspond to the central locations of the anomalies in the model shown in Figure 5, while their boundaries align with the anomalies in the model. The imaging results in Figure 6 demonstrate that SRMAG maintains its high-resolution detection capability for underground anomalies even under the influence of flight attitude variations during surveys in the complex model. Compared with traditional GAFEM rapid imaging methods, when the three-component magnetic field does not have a positive angle error, SRMAG eliminates the impact of flight attitude from the perspective of imaging parameter calculation, exhibiting significantly enhanced resistance to environmental interference.

## 4. Discussion

### 4.1. Fast Imaging Capability

The SRMAG method proposed in this paper is a fast imaging method suitable for real-time field detection. This method achieves a balance between computational time cost and imaging accuracy. Compared with data interpretation techniques such as inversion, the imaging accuracy of the proposed method is somewhat inferior; however, the imaging speed is significantly improved. To more intuitively demonstrate the advantages of the SRMAG method in terms of imaging speed, the time consumed by this method and by inversion are quantitatively compared.

In a complete imaging process, the SRMAG method requires *n* times of 1D forward modeling to obtain the background resistivity, following the procedure of Liu et al. [14], one time of 3D forward modeling, and one calculation of the SRMAG, with the computation time of the SRMAG calculation being negligible. Let the time required for a single 1D forward modeling be denoted as *t*_1_, and that for a single 3D forward modeling as *t*_2_. Accordingly, the total time for one complete imaging process using the SRMAG method is *nt*_1_ + *t*_2_. For inversion, a complete imaging process requires *m* times of 3D forward modeling along with other iterative parameter updates, during which the computation time for parameter iterations is negligible compared with that for 3D forward modeling. Therefore, the time required for one inversion is mt_2_. For GAFEM, 1D forward modeling can be performed using analytical methods, whereas 3D forward modeling can currently only be carried out via numerical simulation methods such as the finite element method. Taking a computer equipped with an Intel I7-12700KF CPU as an example, the time required for one 1D forward modeling for the model in Figure 1 is *t*_1_ = 15 s, while the time for one 3D forward modeling using the commercial finite-element software COMSOL 5.6 [15,16] is *t*_2_ = 2 min 46 s, indicating that *t*_2_ ≫ *t*_1_. In this case, compared with inversion, the imaging time that can be saved by the SRMAG method is (*m* − 1)*t*_2_ − *nt*_1_. Moreover, as the model complexity increases, *t*_2_ increases significantly. For instance, for the model in Figure 5, *t*_2_ increases to 1 h 10 min 44 s, whereas t_1_ remains essentially unchanged because 1D forward modeling does not account for factors such as topographic variations. Consequently, the difference in time consumption between the two imaging approaches increases substantially.

Based on the above analysis, the SRMAG method can be considered to possess fast imaging capability. Furthermore, in practical exploration, if the background resistivity can be obtained before the survey through alternative means, or if a database of background magnetic fields can be established by performing 3D forward modeling for survey areas with different background resistivity values, the imaging efficiency of the method can be further enhanced, thereby improving the capability for real-time field detection.

### 4.2. Influence of Background Resistivity Fitting Error

Although the imaging results presented in Figure 4 and Figure 6 indicate that the proposed SRMAG method can mitigate the influence of flight attitude on the survey and effectively identify anomalous bodies, the imaging results in Figure 6 are not entirely consistent with the simulation model. Through analysis, it is found that the cause of this discrepancy lies in the fact that the background resistivity fitting method adopted by Liu et al. [8] is essentially based on GAFEM 1D forward modeling and obtains the background resistivity through iterative inversion. Although Liu et al. pointed out that relatively regular topographic relief and regular subsurface anomalies do not significantly affect the fitting results [14], for the model in Figure 5, the topographic relief is more complex, and the transmitter source is not an ideal long straight wire. In this case, the background resistivity fitting method employing GAFEM 1D forward modeling already exhibits a considerable geometrical deviation from the known model. Meanwhile, the anomalies set in the Figure 5 model are not regularly distributed rectangular anomalous bodies, which further exacerbates the errors inherent in the 1D forward modeling. This error leads to a significant inaccuracy in the background resistivity obtained by the iterative inversion, which in turn introduces errors into the background magnetic field derived from the 3D forward modeling and ultimately affects the SRMAG calculation results. The existence of this issue has also been demonstrated in studies related to the SMGA method [17].

The background resistivity acquisition method of Liu et al. is adopted in this study in order to enable the complete imaging workflow to be performed using only the measured magnetic field data without recourse to external data in actual field surveys, thus allowing the SRMAG method to serve as a self-contained imaging approach. To enhance the practical applicability of the SRMAG method in field exploration, where external data are available, high-precision background resistivity can be obtained through existing geological data or ground electrode measurements, thereby improving the imaging accuracy of the SRMAG method. Furthermore, achieving high-precision fitting of the background resistivity under complex geological models using scalar magnetic field data constitutes one of the primary future research directions of this study.

### 4.3. Influence of GAFEM Instrument Error

In ideal conditions, since the SRMAG method employs scalar magnetic field data, attitude noise introduced to the three-component magnetic field sensors by the airborne platform at arbitrary angles can be eliminated during the synthesis of the scalar magnetic field from the three-component magnetic field, i.e., in the calculation of (7). However, in field detections, commonly used three-component magnetic field sensors inevitably exhibit orthogonality errors [18,19], which can introduce additional magnetic field components during the synthesis of the scalar magnetic field, thereby causing errors in the scalar magnetic field measurements. This will fundamentally affect the imaging accuracy of the SRMAG method. Furthermore, the GAFEM instrument may also exhibit systematic errors, including scaling factor error, zero bias, inter-axis coupling, and synchronization error. Similar to orthogonality errors, such systematic errors would fundamentally compromise the accuracy of scalar magnetic field measurements and inevitably degrade the imaging performance of the SRMAG method.

Therefore, to ensure that the SRMAG method can achieve satisfactory application performance in field detections, the development of a high-precision GAFEM scalar magnetic field measurement system, as well as systematic error analysis and calibration, will constitute one of the primary future research directions of this study.

## 5. Conclusions

Due to its configuration involving ground-based transmitting and airborne data acquisition, GAFEM inevitably incorporates attitude noise in the collected data. To fundamentally eliminate the influence of flight attitude on GAFEM, this paper proposes a rapid imaging method based on the scalar magnetic field. This approach utilizes attitude-independent scalar magnetic field data as input and combines it with the high-resolution imaging characteristics of gradient fields to compute the scalar magnetic anomaly gradient as the imaging parameter. When the three-component magnetic field does not have a positive angle error, the method theoretically eliminates the influence of flight attitude on GAFEM while enabling efficient detection of underground anomaly locations and boundaries. In this study, the effectiveness of the proposed method is preliminarily validated using a relatively complex simulation model. However, owing to fitting errors in the background resistivity and possible orthogonality errors of the three-component magnetic field sensors in field surveys, the effectiveness and imaging accuracy of the method still require further validation when applied to complex geological structures in practice. In future work, the primary research direction will be to focus on the practical application of the method and to analyze the sources of errors.

## Data Availability

Dataset available on request from the authors.

## Abbreviations

The following abbreviations are used in this manuscript:

GAFEM	Ground-Airborne Frequency-Domain Electromagnetic
ARI	Apparent Resistivity Imaging
SMGA	Space Magnetic Gradient Anomaly
SRMA	Scalar Magnetic Anomaly
SRMAG	Scalar Magnetic Anomaly Gradient
